# Exploiting the Autozygome to Support Previously Published Mendelian Gene-Disease Associations: An Update

**DOI:** 10.3389/fgene.2020.580484

**Published:** 2020-12-31

**Authors:** Sateesh Maddirevula, Hanan E. Shamseldin, Amy Sirr, Lama AlAbdi, Russell S. Lo, Nour Ewida, Mashael Al-Qahtani, Mais Hashem, Firdous Abdulwahab, Omar Aboyousef, Namik Kaya, Dorota Monies, May H. Salem, Naffaa Al Harbi, Hesham M. Aldhalaan, Hamad Alzaidan, Hadeel M. Almanea, Abrar K. Alsalamah, Fuad Al Mutairi, Samira Ismail, Ghada M. H. Abdel-Salam, Amal Alhashem, Ali Asery, Eissa Faqeih, Amal AlQassmi, Waleed Al-Hamoudi, Talal Algoufi, Mohammad Shagrani, Aimée M. Dudley, Fowzan S. Alkuraya

**Affiliations:** ^1^Department of Genetics, King Faisal Specialist Hospital and Research Center, Riyadh, Saudi Arabia; ^2^Pacific Northwest Research Institute, Seattle, WA, United States; ^3^Department of Zoology, College of Science, King Saud University, Riyadh, Saudi Arabia; ^4^Pediatric Nephrology Service, Department of Pediatrics, King Faisal Specialist Hospital and Research Center, Jeddah, Saudi Arabia; ^5^Department of Neuroscience, King Faisal Specialist Hospital and Research Center, Riyadh, Saudi Arabia; ^6^Department of Medical Genetics, King Faisal Specialist Hospital and Research Center, Riyadh, Saudi Arabia; ^7^College of Medicine, Alfaisal University, Riyadh, Saudi Arabia; ^8^Anatomic Pathology, King Faisal Specialist Hospital and Research Center, Riyadh, Saudi Arabia; ^9^Vitreoretinal and Uveitis Divisions, King Khaled Eye Specialist Hospital, Riyadh, Saudi Arabia; ^10^Medical Genetics Division, Department of Pediatrics, King Abdullah International Medical Research Centre, King Abdulaziz Medical City, King Saud Bin Abdulaziz University for Health Sciences, Riyadh, Saudi Arabia; ^11^Human Genetics & Genome Research Division, Clinical Genetics Department, Center of Excellence of Human Genetics, National Research Centre, Cairo, Egypt; ^12^Department of Pediatric, Prince Sultan Medical Military City, Riyadh, Saudi Arabia; ^13^Section of Pediatric Gastroenterology, Children’s Specialist Hospital, King Fahad Medical City, Riyadh, Saudi Arabia; ^14^Department of Pediatric Subspecialties, Children’s Hospital, King Fahad Medical City, Riyadh, Saudi Arabia; ^15^Pediatric Neurology, King Saud Medical City, Riyadh, Saudi Arabia; ^16^Department of Medicine, College of Medicine, King Saud University, Riyadh, Saudi Arabia; ^17^King Faisal Specialist Hospital and Research Center, Organ Transplant Centre, Riyadh, Saudi Arabia

**Keywords:** autozygosity, diabetes, yeast, founder mutation, disease-gene associations

## Abstract

There is a growing interest in standardizing gene-disease associations for the purpose of facilitating the proper classification of variants in the context of Mendelian diseases. One key line of evidence is the independent observation of pathogenic variants in unrelated individuals with similar phenotypes. Here, we expand on our previous effort to exploit the power of autozygosity to produce homozygous pathogenic variants that are otherwise very difficult to encounter in the homozygous state due to their rarity. The identification of such variants in genes with only tentative associations to Mendelian diseases can add to the existing evidence when observed in the context of compatible phenotypes. In this study, we report 20 homozygous variants in 18 genes (*ADAMTS18, ARNT2, ASTN1, C3, DMBX1, DUT, GABRB3, GM2A, KIF12, LOXL3, NUP160, PTRHD1, RAP1GDS1, RHOBTB2, SIGMAR1, SPAST, TENM3*, and *WASHC5*) that satisfy the ACMG classification for pathogenic/likely pathogenic if the involved genes had confirmed rather than tentative links to diseases. These variants were selected because they were truncating, founder with compelling segregation or supported by robust functional assays as with the *DUT* variant that we present its validation using yeast model. Our findings support the previously reported disease associations for these genes and represent a step toward their confirmation.

## Introduction

Mendelian (aka monogenic) diseases are collectively common despite the rarity of most individual entities. Their diagnostic landscape has been fundamentally changed by massively parallel sequencing technologies. These technological advances have enabled the rapid and high throughput screening of a large number of genes thus circumventing the historical bottleneck of sequential sequencing of genes deemed relevant to the clinical phenotype. In the case of exome or genome sequencing, an additional advantage lies in their potential to detect causal variants in genes with no established disease links in humans, i.e., novel candidates (Alkuraya, 2016). The latter scenario poses a major challenge because variants in such genes cannot be classified according to the current ACMG guidelines as pathogenic or likely pathogenic so these patient’s molecular diagnosis remains ambiguous until such time that sufficient evidence is established in the literature to confirm the disease-gene association (Richards et al., 2015). The widespread use of exome and genome sequencing clinically has made this problem more acute.

As noted by the ClinGen guidance, there are two major lines of evidence to support gene-disease association: genetic (e.g., case series) and experimental (e.g., animal model) (Strande et al., 2017). To reflect the superiority of genetic evidence, the maximum allowed score for the experimental evidence (6 points) is only half of that allowed for genetic evidence (12 points). This underscores the importance of reporting additional cases with compatible phenotypes to increase the confidence of gene-disease associations. In the case of candidate genes for autosomal recessive phenotypes, consanguineous populations offer a unique opportunity to accelerate the confirmation of these candidates (Alkuraya, 2012, 2016). The enrichment of autozygosity in consanguineous populations, as measured by the inbreeding coefficient, translates into a rich supply of homozygous variants including deleterious variants (Alkuraya, 2010). If these deleterious variants involve previously reported candidate genes and the observed phenotype is compatible, this provides an important genetic evidence especially when the variant is predicted null or segregates strongly in a multiplex family as stipulated by ClinGen.

We have previously implemented this approach to support the candidacy of dozens of previously reported candidate genes (Maddirevula et al., 2019b). This study is a continuation of that effort where we present supportive genetic evidence of 18 additional genes with only tentative gene-disease associations.

## Materials and Methods

### Human Subjects

Informed consent was obtained from all subjects included in this analysis in accordance with the local IRB guidelines (KFSHRC RAC# 2070 023, 2080 006, 2121 053). Phenotypic information was collected, and segregation analysis was performed where applicable among available relatives.

### Autozygome Mapping, Exome Analysis, and Variant Calling

Exome analysis and variant filtering of variants by autozygome analysis was as described before (Anazi et al., 2017; Monies et al., 2019). Briefly variants were retained only if coding/splice, within autozygome, novel or very rare (MAF <0.001) in our internal database (SHGP database with 2,379 exomes) and gnomAD. HGMD reported variants and genes with OMIM entry were prioritized in the analysis. *In silico* (CADD, PolyPhen, SIFT, and TraP) pathogenicity was considered for the all variants.

We only included in this report variants that met both of the following criteria:

1- Variant that would have met the ACMG guidelines for pathogenic/likely pathogenic if the involved genes were to have an established association with the phenotype.
2- Variant in a gene with less than definitive link to phenotype because (a) gene has no listed OMIM phenotype, (b) gene has a listed OMIM phenotype but with a question mark, (c) gene has a listed OMIM phenotype based on a single study, or (d) variant with incompatible mode of inheritance to that reported for the respective gene in OMIM.

### Yeast Experimental Methods

Unless noted, all *Saccharomyces cerevisiae* strains were grown at 30°C using standard media conditions and methods (Rose et al., 1990). Strains are derived from a prototrophic diploid strain (Winston et al., 1995) modified to bear a single copy of the *DUT* ortholog, *DUT1*, which is an essential yeast gene (*dut1*Δ0/*DUT1*) (Gadsden et al., 1993; Winzeler et al., 1999; Supplementary Table S1).

The allele *yDUT* refers to sequence encompassing 293 bp of the *S. cerevisiae DUT1* promoter region, the human *DUT* protein coding sequence (NP_001939.1), and 202 bp of the yeast *DUT1* terminator sequence. The protein coding sequence was codon optimized for expression in yeast (IDT). gBlocks of the *yDUT* and *yDUT-R128Q* alleles were synthesized (IDT) with added homology to plasmid AB523. Constructs were then assembled (NEB HiFi Assembly) into *Afe*I digested AB523 to generate plasmids AB527 and AB531 (Supplementary Table S2). For control strains, the native S. cerevisiae *DUT1* allele, including the same regulatory regions as yDUT was PCR amplified from yeast DNA using primers with added homology to AB523 using primers DUT1_HO_F and DUT1_natNT1_R (Supplementary Table S3) and assembled into *Afe*I digested AB523 to create plasmid AB525. All plasmids contain targeting sequence to direct integration of the construct to a neutral location (the *HO* locus) in the yeast genome on chromosome IV of the yeast genome (Voth et al., 2001).

*Not*I digested AB525, AB527, and AB531 were transformed into a *dut1*Δ0/*DUT1* diploid (YAD714 and YAD715). Spores bearing drug markers for both the *dut1*Δ0 locus and the desired *yDUT* or *DUT1* construct were isolated by standard tetrad dissection and confirmed by PCR and Sanger’s sequencing (Supplementary Table S1). Six isolates of each genotype were grown overnight in liquid cultures (YPD 2% glucose) and then spotted onto agar plates (YPD 2% glucose). Plates were imaged after 2 days of growth at 30°C. Yeast growth was quantified as described previously (Sirr et al., 2020).

Plasmid sequences are available in GenBank (Accession numbers in Supplementary Table S1). All yeast strains and plasmids are available upon request (aimee.dudley@gmail.com).

## Results

We report 24 patients who harbor 20 homozygous variants that met our inclusion criteria and spanned 18 genes (Table 1). Clinical details of all included families in this study are provided in Supplementary Table S4. Detailed pedigrees of the families with the segregation data are provided in Supplementary Figure S1. These variants can be grouped into three classes listed below along with all the cases contained therein:

**TABLE 1:** List of the identified homozygous variants in 18 genes.

**Case ID**	**Phenotype**	**Gene**	**Variant**	**ACMG classification**	**Previously reportred phenotype (OMIM/Reference)**
20DG0576	Microcornea and myopic chorioretinal atrophy	*ADAMTS18*	NM_199355.2:c.1298C> A;p.(Thr433Asn)	Likely pathogenic (PM2; PP3; PP4; PP5; BP1)	Microcornea, myopic chorioretinal atrophy, and telecanthus (OMIM: 615458)
20DG0577	Failure to thrive, microcephaly, developmental delay, and poor vision	*ARNT2*	NM_014862.3:c.147-1G>A	Pathogenic (PVS1; PM2; PP3)	?Webb-Dattani syndrome (OMIM:615926)
20DG0578	Septo-optic dysplasia, developmental delay, short stature, and microcephaly	*ARNT2*	NM_014862.3:c.147-1G>A	Pathogenic (PVS1; PM2; PP3)	?Webb-Dattani syndrome (OMIM:615926)
20DG1278	Autism, ADHD, and seizures	*ASTN1*	NM_001286164.2:c.3159_ 3160del;p.(Gln1053Hisfs*13)	Pathogenic (PVS1; PM1; PM2)	Brain malformation and spastic tetraplegia, epilepsy and developmental delay (PMID: 26539891/29706646)
20DG0579	Atypical hemolytic uremic syndrome	*C3*	NM_000064.2:c.3343G> A;p.(Asp1115Asn)	Likely pathogenic (PM1; PM2; PP3; PP5; BP1)	{Hemolytic uremic syndrome, atypical, susceptibility to, 5}, Autosomal dominant (OMIM:612925)
20DG0580	Global developmental delay, seizures, and hypotonia	*DMBX1*	NM_147192.2:c.367C> T;p.(Arg123Trp)	Likely pathogenic (PM1; PM2; PP3; PP5)	Global developmental delay, epilepsy, hypotonia, hearing loss, hyperopia, and strabismus (PMID:25558065)
09DG01598	Pancytopenia	*DUT*	NM_001025248.1:c.647G> A;p.(Arg216Gln)	Likely pathogenic (PS3; PM2; PP3)	Novel monogenic syndrome of diabetes and bone marrow failure (PMID:28073829
20DG0582	Epilepsy and global developmental delay	*GABRB3*	NM_001191320.1:c.890C> G;p.(Ser297*)	Likely pathogenic (PM1; PM2; PP2; PP3)	Developmental and epileptic encephalopathy 43, Autosomal dominant (OMIM:617113)
20DG0583	Distinct progressive chorea-dementia syndrome	*GM2A*	NM_000405.5:c.164C>T;p. (Pro55Leu)	Likely pathogenic (PM1; PM2; PP3; PP5; BP1)	GM2-gangliosidosis, AB variant (OMIM: 272750)
20DG0584	High GGT cholestasis	*KIF12*	NM_138424.1:c.610G>A;p. (Val204Met)	Likely pathogenic^#^ (PM2, PP1, PP3, PP5)	Cholestasis with high gamma-glutamyltransferase (PMID:30250217)
20DG0585	High GGT cholestasis	*KIF12*	NM_138424.1:c.463C>T;p. (Arg155*)	Pathogenic (PVS1; PM2; PP3)	Cholestasis with high gamma-glutamyltransferase (PMID:30250217)
20DG0586	High GGT cholestasis	*KIF12*	NM_138424.1:c.290A>G;p. (His97Arg)	VUS (PM2; PP3)	Cholestasis with high gamma-glutamyltransferase (PMID:30250217)
20DG0587	High GGT cholestasis	*KIF12*	NM_138424.1:c.463C>T;p. (Arg155*)	Pathogenic (PVS1; PM2; PP3)	Cholestasis with high gamma-glutamyltransferase (PMID:30250217)
20DG1038	Cataract, retinal detachment, high myopia, intellectual disability, and chronic constipation	*LOXL3*	NM_032603.4:c.824dup;p. (Ala277Cysfs*57)	Pathogenic (PVS1; PM2; PP3)	Stickler syndrome and high myopia (PMID:25663169 and 26957899)
18DG0487	Developmental delay, epilepsy, and nephrotic syndrome	*NUP160*	NM_015231.1:c.1179+5G>A	Pathogenic (PS3; PM2; PM4; PP1; PP3)	?Nephrotic syndrome, type 19 (OMIM:618178)
15DG1365	Intellectual disability	*PTRHD1*	NM_001013663.1:c.365G> A;p.(Arg122Gln)	Likely pathogenic^#^ (PM2; PP1; PP3; PP5; BP4)	Intellectual disability (PMID:27134041)
10DG0745	Intellectual disability	*PTRHD1*	NM_001013663.1:c.365G> A;p.(Arg122Gln)	Likely pathogenic^#^ (PM2; PP1; PP3; PP5; BP4)	Intellectual disability (PMID:27134041)
13DG0792*	Global developmental delay and Caroli disease	*PTRHD1*	NM_001013663.1:c.365G> A;p.(Arg122Gln)	Likely pathogenic^#^ (PM2; PP1; PP3; PP5; BP4)	Intellectual disability (PMID:27134041)
20DG0588**	Developmental delay and dysmorphism	*RAP1GDS1*	NM_001100426.1:c.1444-1G>A	Pathogenic (PVS1; PM2; PP3)	Intellectual disability, GDD and hypotonia (PMID:27134041)
20DG0038	Global developmental delay, no epilepsy	*RHOBTB2*	NM_001160036.2:c.460C> T:p.(Arg154*)	Pathogenic (PVS1; PM2; PP3)	Epileptic encephalopathy, early infantile, 64, Autosomal dominant (OMIM:618004)
20DG0589	Spastic paraplegia	*SIGMAR1*	NM_005866.2:c.73del G;p.(Val25Serfs*18)	Pathogenic (PVS1; PM2; PP3)	?Spinal muscular atrophy, distal, autosomal recessive, 2 (OMIM:605726)
20DG1269	Developmental regression, optic atrophy, hypotonia, spasticity, and seizures	*SPAST*	NM_199436.2:c.1290A> T;p.(Lys430Asn)	Pathogenic (PS1, PM1, PM2, PP2, PP3)	Spastic paraplegia 4, autosomal dominant (OMIM:182601)
16DG1417	Microcornea and coloboma	*TENM3*	NM_001080477.1:c.6006_ 6009del;p.(Gln2003Phefs*10)	Pathogenic (PVS1; PM2; PP3)	Microphthalmia, isolated, with coloboma 9 (OMIM:615145)
09DG00555	Craniosynostosis and dysmrophic features	*WASHC5*	NM_014846.3:c.2849A> G;p.(Lys950Arg)	Likely pathogenic (PM2; PP2; PP3)	Ritscher-Schinzel syndrome 1 (OMIM:220210)

*Patient with a dual diagnosis. **Published elsewhere. ^#^PP1 is used as a stronger evidence due to the ancient founder nature of the variant.

**FIGURE 1:**
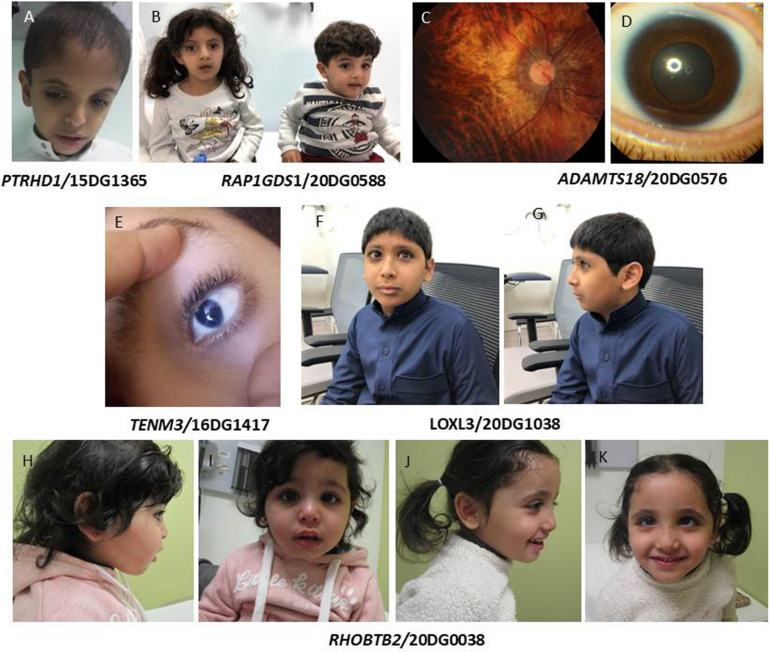
**(A)** Subject with *PTRHD1* mutation lacking gross facial dysmorphism. **(B)** Siblings with *RAP1GDS1* mutation showing small mouth, prominent nasal bridge, mild synophrys, and micrognathia. **(C,D)** Color fundus photo of the right eye showing myopic chorioretinal atrophy and slit-lamp photograph of the left eye showing microcornea of the patient with *ADAMTS18* mutation. **(E)** Patient with mutation in *TENM3* showing microcornia. **(F,G)** Facial images showing high myopia in a patient with mutation in *LOXL3.* **(H–K)** Facial images of siblings with homozygous mutation in *RHOBTB2* showing depressed nasal bridge and strabismus.

Class 1: variants that are identical to the ones that were the basis for the original report of candidacy. Since these are different patients from those originally reported, they serve as a strong line of segregation-based evidence according to the ACMG guidelines. For example, *PTRHD1*:NM_001013663.1:c.365G>A;p.(Arg122Gln) was reported in 2017 (Reuter et al., 2017) in an Egyptian family with mild intellectual disability (ID) prompting the authors to propose *PTRHD1* as a novel candidate gene. Here we report three unrelated Saudi patients with the same variant and confirm its founder nature based on haplotype analysis. All three patients had ID with no facial dysmorphism (Figure 1A). Of note, a dual molecular diagnosis is observed in the patient 13DG0792, who is homozygous for the founder variant *PTRHD1* and a ciliopathy phenotype (Caroli disease) caused by the variant *WDR35* [NM_001006657.1:c.206G>A; p.(Gly69Asp)] as described before (Shaheen et al., 2016).

Another example is the founder *KIF12* variants NM_138424. 1:c.610G>A;p.(Val204Met) and NM_138424.1:c.463C>T;p. (Arg155^∗^) that we previously published in patients with congenital hepatic fibrosis/sclerosing cholangitis and high gamma-glutamyltransferase (GGT)-cholestasis (Maddirevula et al., 2019a), which we identified in another patient with the same phenotype. Of note, we also identified in this study a novel homozygous variant [*KIF12*:NM_ 138424.1:c.290A>G;p.(His97Arg)] in a patient with a late-onset phenotype (17 years) although we emphasize that this remains VUS at this point. The founder variant we identified in *RAP1GDS1* (NM_001100426.1:c.1444-1G>A) is another example. This variant was reported in an apparently new syndrome of dysmorphic facies and intellectual disability (Asiri et al., 2020). We identified the same variant in the two siblings with the same phenotype (Figure 1B) (Bertoli-Avella et al., submitted).

*ADAMTS18* has a listed OMIM phenotype (microcornea, myopic chorioretinal atrophy, and telecanthus, MMCAT) based on a previous study in which we reported several families with different homozygous variants (Aldahmesh et al., 2013); however, our finding has not been confirmed by follow up studies. Here, we report the identification of a new case with the same founder variant [NM_001326358.2:c.782C>A;p.(Thr261Asn)] who, in addition to the classical findings of microcornea, myopic chorioretinal atrophy and telecanthus (Figures 1C,D), also has epilepsy and hypertension that may or may not be related to congenital renal anomalies. Interestingly, we have previously argued in support of the involvement of *DMBX1* in the etiology of autosomal recessive intellectual disability based on the original family and a follow up family with the same founder (NM_147192.2:c.367C>T:p.(Arg123Trp) (Maddirevula et al., 2019b). However, this gene remains with no OMIM phenotype. Here, we report a new family with the same founder and same phenotype (Table 1).

*GM2A* is an established gene for GM2-gangliosidosis, AB variant (MIM: 272750). An extended multiplex family was reported with a surprisingly different phenotype that lacks organomegaly and cherry red macula and instead comprises childhood onset progressive chorea-dementia syndrome, which was proposed as a novel *GM2A*-related phenotype (Alazami et al., 2015;Salih et al., 2015). We identified the same founder variant [NM_000405.5:c.164C>T;p.(Pro55Leu)] in a new patient with an identical phenotype (Table 1). The last example in this category is *C3*:NM_000064.2:c.3343G>A;p.(Asp1115Asn), which we report here for the first time in homozygosity even though it was reported twice in the heterozygous state in patients with atypical hemolytic uremic syndrome (Fremeaux-Bacchi et al., 2008;Schramm et al., 2015), just as observed in the patient we report here. Unfortunately, the ancestry of the two previously reported patients has not been described so we are unable to speculate on its potential founder nature.

**FIGURE 2:**
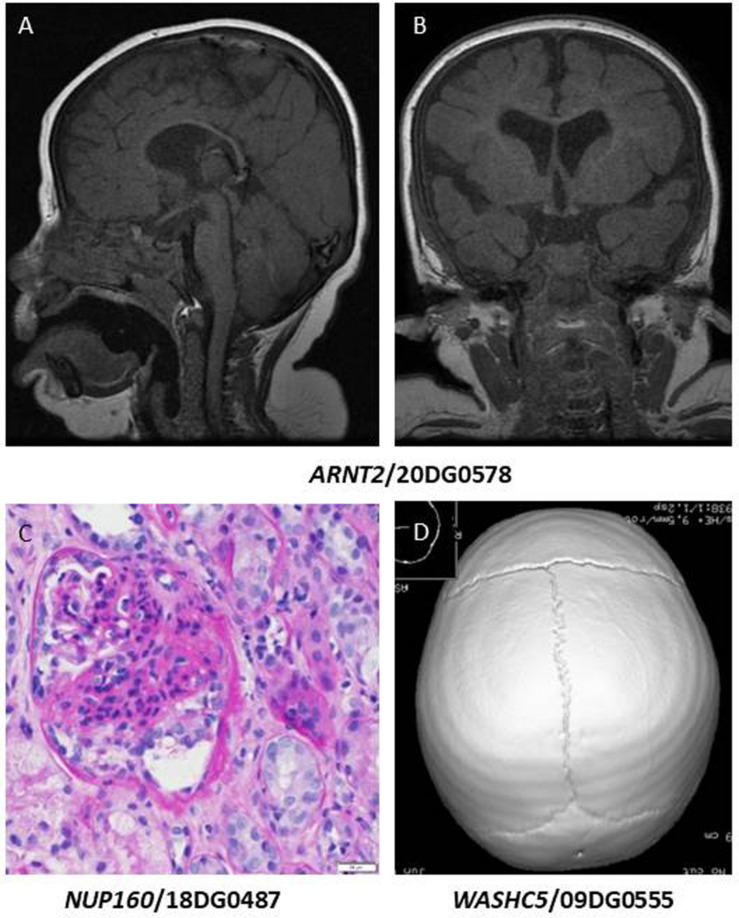
**(A,B)** Brain MRI images of the patient with homozygous *ARNT2* mutation showing thinning of the corpus callosum, brain atrophic changes, and hypoplasia of the pituitary gland with no demonstrable posterior lobe bright spot or pituitary stalk. **(C)** Renal biopsy of the patient with *NUP160* mutation showing segmental solidification of the glomerular tuft (classical segmental scar). **(D)** CT scan image of the patient with a variant in *WASHC5* showing partial premature closure of the lambdoid suture.

Class 2: variants that expand the allelic heterogeneity of the gene-disease association. These include two unrelated patients who share a novel founder variant (as revealed by haplotype sharing) *ARNT2*:NM_014862.3:c.147-1G>A. We had proposed *ARNT2* as a novel candidate gene for a condition characterized by hypothalamo-pituitary-frontotemporal hypoplasia with visual and renal anomalies based on a single frameshift variant in a multiplex family (Webb et al., 2013). Brain MRI for one of these two patients showed hypoplasia of the pituitary gland with no demonstrable posterior lobe or pituitary stalk and brain atrophic changes with delayed myelination (Figures 2A,B), which is consistent with the previously reported family. The canonical splicing founder variant in *ARNT2* in the two siblings we report here lends further support to the original report.

Another example is *TENM3* (formally ODZ3), which we had proposed as a novel gene for colobomatous microphthalmia based on a single frameshift variant in a multiplex family (Aldahmesh et al., 2012). Here, we report a novel homozygous frameshift variant [NM_001080477.1:c.6006_6009del;p.(Gln2003Phefs^∗^10)] in a patient with a similar phenotype (Figure 1E).

Similarly, *NUP160* is a gene we had proposed as a novel candidate for steroid-resistant nephrotic syndrome based on two siblings who were compound heterozygous for missense and nonsense variants (Braun et al., 2018). The variant we report here (NM_015231.1:c.1179+5G>A) was confirmed by RTPCR to cause abnormal splicing [r.1102_1179del;p.(Phe368_Gln393del)] (Maddirevula et al., 2020). The patient has steroid-resistant nephrotic syndrome and chronic kidney disease, which is consistent with the previously reported patients (Figure 2C). However, we note the additional neurological features of intellectual disability and epilepsy, which co-segregated with the steroid-resistant nephrotic syndrome in both siblings.

Other variants in this class include *SIGMAR1*:NM_005866.2:c.73delG;p.(Val25Serfs^∗^18), which supports the involvement of this gene in autosomal recessive juvenile amyotrophic lateral sclerosis (Table 1). *ASTN1* was reported as a candidate gene for brain malformation (Karaca et al., 2015), and spastic tetraplegia, epilepsy and developmental delay (Wiszniewski et al., 2018). Here we report a homozygous truncating variant [*ASTN1*:NM_001286164.2:c.3159_3160del;p.(Gln1053Hisfs^∗^13)] in a patient with autism, ADHD and seizures. *LOXL3* was reported in patients with high myopia (Li et al., 2016) and in Sticklers syndrome (Alzahrani et al., 2015). Interestingly, we report a truncating variant in *LOXL3* [NM_032603.4:c.824dup;p.(Ala277Cysfs^∗^57)] in a patient with high myopia (Figures 1F,G), retinal detachment with mild ID (IQ 62). It is unclear, however, if ID is related to the *LOXL3* variant. To date a single report was published linking *WASHC5* to variable craniofacial features, cerebellar and cardiac anomalies (Ritscher-Schinzel syndrome 1/MIM: 220210). We report a homozygous exonic splicing variant in *WASHC5* [NM_014846.3:c.2849A>G;p.(Lys950Arg)] in a patient with a distinct clinical feature of lambdoid synostosis (Figure 2D) along with dysmrophic features like malar hypoplasia, micrognathia, mild turricephaly, and broad hallux.

**FIGURE 3:**
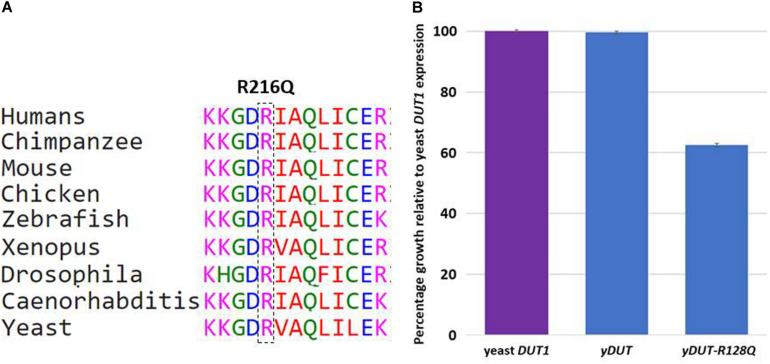
**(A)** Conservation of variant (p.Arg216Gln) in DUT. **(B)** Yeast assays of human DUT variant suggest partial loss of function for the human Arg216Gln equivalent, yDUT-R128Q. Values represent the mean and standard error of the mean for each genotype, rescaled to make the wildtype yDUT mean value equal to 1. The purple bar is the growth value of the control strains expressing the yeast ortholog *DUT1*. Blue bars are growth values of yeast strains expressing either the wildtype yDUT construct or the same construct harboring the R128Q variant. Error bars are standard error of the mean. Assays were performed in replicate with 6 independently constructed clones each assayed 12 times.

The last variant in this class is worth highlighting to justify its inclusion despite being missense. *DUT* was published as a candidate for a novel syndromic form of diabetes involving bone marrow failure based on a single missense variant (Dos Santos et al., 2017). The patient we present here has the following novel missense variant *DUT*:NM_001025248.1:c.647G>A;p.(Arg216Gln), which we included because of additional functional evidence in yeast. Arginine at 216 is highly conserved from human to yeast (Figure 3A). The yeast and human orthologs are highly conserved (55% amino acid identity across the length of the protein) (Tchigvintsev et al., 2011) and a previous publication had shown that the human ortholog is able to functionally replace (genetically complement) a deletion of the yeast gene (Hamza et al., 2015; Kachroo et al., 2015). Because the yeast Dut1 protein localizes to the nucleus and cytoplasm (Huh et al., 2003), we used the *DUT* protein sequence corresponding to the nuclear isoform *DUT-N* (NP_001939.1), which lacks the 93 amino acid mitochondrial leader sequence (Uniprot: P33316). As such, our allele yDUT-R128Q, harbors the same amino acid change as NM_001025248.1:c.647G>A;p.(Arg216Gln). Consistent with previous studies (Hamza et al., 2015; Kachroo et al., 2015), our yeast codon optimized version of the *DUT* protein coding sequence integrated into the yeast genome and expressed from the yeast *DUT1* transcriptional promoter (section “Materials and Methods”) was able to completely (100%) complement loss of the yeast gene (Figure 3B). In contrast, the same construct harboring the Arg216Gln allele, exhibited a significant loss of function phenotype, with 63% growth relative to wildtype (Figure 3B).

Class 3: These are variants identified in homozygosity in genes with associations only to dominant phenotypes in OMIM. We opted to include these variants given their important implications in the interpretation of the lack of phenotype among the obligate carrier parents as described before (Monies et al., 2017). The first example is *RHOBTB2*, a gene only linked to autosomal dominant epileptic encephalopathy, early infantile, 64 (MIM: 618004). Here, we report the identification of a homozygous nonsense variant [NM_001160036.2:c.460C>T:p.(Arg154^∗^)] in three siblings with global developmental delay, facial dysmorphism (Figures 1H–K), normal brain MRI and no epilepsy, thus expanding the phenotype in addition to expanding the mode of inheritance of *RHOBTB2*-related neurodevelopmental disease.

*GABRB3* is another example since this is a gene only associated with autosomal dominant epileptic encephalopathy, early infantile, 43. We identified a homozygous nonsense variant [NM_001191320.1:c.890C>G;p.(Ser297^∗^)] in a patient with dystonia and infantile spasm with profound global developmental delay (Table 1).

Another example is *SPAST*, an established gene for autosomal dominant spastic paraplegia 4 (MIM: 182601). We report three siblings with developmental regression, optic atrophy, central hypotonia, peripheral spasticity and seizures who have a pathogenic homozygous missense variant [NM_199436.2:c.1290A>T;p.(Lys430Asn)]. In these three examples, the parents were normal clinically, consistent with the bona fide recessive inheritance we propose here.

## Discussion

The determination of whether a given Mendelian gene-disease association is established has been largely subjective until the publication of an evidence-based framework by ClinGen, which represents a major step toward standardization (Strande et al., 2017). For example, the framework proposes replacing the binary “candidate” vs. “established” with a much more nuanced labeling system that more truly reflects the spectrum of evidence. The supporting evidence, when present, is classified into definitive, strong, moderate and limited. Importantly, the framework also accounts for evidence that contradicts previously reported gene-disease associations rendering the latter disputed or even refuted. Indeed, we have previously shown the power of the autozygome to generate pathogenic variants in individuals that lack phenotypes previously associated with the respective genes (Shamia et al., 2015). However, our focus in this study is on variants that lend supportive evidence.

The current OMIM listing of Mendelian phenotypes does not necessarily reflect the ClinGen framework since it fails to include genes with definitive evidence e.g., *LBR*, *MYO9A*, and *VPS8* while including others with only limited evidence even without the cautionary question mark e.g., *PCK2* has a listed OMIM phenotype even though no single patient with *PCK2* mutation has been reported to date (Stark and Kibbey, 2014). Nonetheless, we have opted to use OMIM as a starting point given its authoritative standing in the clinical genetics community, and the genes we report in this study, as originally intended, only have “moderate” (e.g., *TENM3, SIGMAR1, ARNT2*, and *C3*) or “limited” (e.g., *NDUFA12* and *NUP160*) evidence supporting their involvement in human diseases leaving room for further support as we hope to have accomplished through our analysis. Because our goal in this paper is to enhance the interpretation of variants, we opted to also include recessive variants in genes with links only to dominant phenotypes. Establishing bona fide recessive inheritance of these genes greatly influences the interpretation of apparently pathogenic variants in asymptomatic heterozygous individuals who would otherwise be considered as non-penetrant individuals. The impact this change of interpretation has on recurrence risk estimates cannot be overemphasized.

It should be noted that the variants we report do not necessarily make the respective gene-disease associations “definitive.” Instead, they corroborate the previously published association and can be considered collectively with future reported evidence in an iterative process as outlined by the ClinGen framework. Nonetheless, clinical molecular laboratories will find this and similar reports helpful in their determination of genes that are relevant to the tested patient’s phenotype. One should also note that a given gene may have several disease associations and the evidence for each must be weighted differently. For example, while the association between C3 and C3 deficiency has “definitive” supportive evidence, C3-atypical hemolytic uremic syndrome association has “limited” supportive evidence; hence the value of the variant presented in this study.

Although the supportive evidence presented in this study is overwhelmingly genetic in nature, we also share experimental evidence in support of the pathogenesis of the missense variant identified in *DUT*. Since the case reported by Dos Santos et al. (2017), no follow up studies have been published on the involvement of *DUT* in the pathogenesis of the syndrome of diabetes with bone marrow failure. Without functional validation, the novel missense variant we encountered in a patient with that syndrome will not have much weight due to lack of compelling segregation. However, the strong conservation of this gene in yeast presented an opportunity to validate its deleterious effect on the gene function, which strengthens its relevance as a supportive evidence. Indeed, the use of yeast as a model organism as a high throughput system for testing VUS in genes that are conserved has seen increasing use in recent years (Hamza et al., 2015; Sun et al., 2016; Sirr et al., 2020).

In summary, we present data that support 18 previously reported gene-disease associations. This approach also allowed us to identify homozygous pathogenic variants in autosomal dominant genes like *RHOBTB3*, *SPAST*, and *GABRB3.* The identification of these variants in the homozygous state despite their rarity is an obvious advantage of conducting this kind of analysis in a highly consanguineous population. The benefits of this approach, however, extend to the global clinical genetics community.

## Data Availability Statement

The datasets for this article are not publicly available due to concerns regarding participant/patient anonymity. Requests to access the datasets should be directed to the FSA FAlKuraya@kfshrc.edu.sa.

## Ethics Statement

The studies involving human participants were reviewed and approved by the Office of Research Ethics at the King Faisal Specialist Hospital & Research Center, Riyadh, Saudi Arabia. Written informed consent to participate in this study was provided by the participants’ legal guardian/next of kin. Written informed consent was obtained from the individual(s), and minor(s)’ legal guardian/next of kin, for the publication of any potentially identifiable images or data included in this article.

## Author Contributions

SM performed the analysis, collected and organized the data, and wrote the manuscript. HS, LA, NK, and DM performed analysis of exomes and validation of variants. MH, OA and FA coordinated with the patient for sampling. NE and MA-Q performed segregation analysis. MHS, NA, HMAld, HA, HMAlm, AKA, FAM, SI, GA-S, AAl, AAs, EF, AA, WA, TA, and MS referred the patients for genetic test and provided phenotypic data. AS performed the yeast validation work. AD supervised the yeast validation work and wrote the manuscript. FSA designed, supervised the project, and wrote the manuscript. All authors contributed to the article and approved the submitted version.

## Conflict of Interest

The authors declare that the research was conducted in the absence of any commercial or financial relationships that could be construed as a potential conflict of interest.

## Supplementary Material

Supplementary Figure S1 Pedigrees and segregation data of all the study families.

Supplementary Table S1 *S. cerevisiae* strains used in this study.

Supplementary Table S2 Plasmids used in this study.

Supplementary Table S3 Oligonucleotides and gBlocks (IDT) used in this study.

Supplementary Table S4 Clinical synopsis of the 24 patients described in this study. ACMG, American College of Medical Genetics; SHGP, Saudi Human Genome Program; HGMD, Human Gene Mutation Database.

## References

[B1] Alazami A. M. Patel N. Shamseldin H. E. Anazi S. Al-Dosari M. S. Alzahrani F. (2015). Accelerating novel candidate gene discovery in neurogenetic disorders via whole-exome sequencing of prescreened multiplex consanguineous families. *Cell Rep.* 10 148–161. 10.1016/j.celrep.2014.12.015 25558065

[B2] Aldahmesh M. A. Alshammari M. J. Khan A. O. Mohamed J. Y. Alhabib F. A. Alkuraya F. S. (2013). The syndrome of microcornea, myopic chorioretinal atrophy, and telecanthus (MMCAT) is caused by mutations in ADAMTS18. *Hum. Mutat.* 34 1195–1199. 10.1002/humu.22374 23818446

[B3] Aldahmesh M. A. Mohammed J. Y. Al-Hazzaa S. Alkuraya F. S. (2012). Homozygous null mutation in ODZ3 causes microphthalmia in humans. *Genet. Med.* 14 900–904. 10.1038/gim.2012.71 22766609

[B4] Alkuraya F. S. (2010). Autozygome decoded. *Genet. Med.* 12 765–771. 10.1097/gim.0b013e3181fbfcc4 21189493

[B5] Alkuraya F. S. (2012). Discovery of rare homozygous mutations from studies of consanguineous pedigrees. *Curr. Protoc. Hum. Genet.* Chapter 6:Unit612. 10.1002/0471142905.hg0612s75 23074070

[B6] Alkuraya F. S. (2016). Discovery of mutations for Mendelian disorders. *Hum. Genet.* 135 615–623. 10.1007/s00439-016-1664-8 27068822

[B7] Alzahrani F. Al Hazzaa S. A. Tayeb H. Alkuraya F. S. (2015). LOXL3, encoding lysyl oxidase-like 3, is mutated in a family with autosomal recessive Stickler syndrome. *Hum. Genet.* 134 451–453. 10.1007/s00439-015-1531-z 25663169

[B8] Anazi S. Maddirevula S. Faqeih E. Alsedairy H. Alzahrani F. Shamseldin H. E. (2017). Clinical genomics expands the morbid genome of intellectual disability and offers a high diagnostic yield. *Mol. Psychiatry* 22 615–624. 10.1038/mp.2016.113 27431290

[B9] Asiri A. Aloyouni E. Umair M. Alyafee Y. Al Tuwaijri A. Alhamoudi K. M. (2020). Mutated RAP1GDS1 causes a new syndrome of dysmorphic feature, intellectual disability & speech delay. *Ann. Clin. Transl. Neurol.* 7 956–964. 10.1002/acn3.51059 32431071 PMC7318102

[B10] Braun D. A. Lovric S. Schapiro D. Schneider R. Marquez J. Asif M. (2018). Mutations in multiple components of the nuclear pore complex cause nephrotic syndrome. *J. Clin. Invest.* 128 4313–4328. 30179222 10.1172/JCI98688 PMC6159964

[B11] Dos Santos R. S. Daures M. Philippi A. Romero S. Marselli L. Marchetti P. (2017). dUTPase (DUT) is mutated in a novel monogenic syndrome with diabetes and bone marrow failure. *Diabetes* 66 1086–1096. 10.2337/db16-0839 28073829

[B12] Fremeaux-Bacchi V. Miller E. C. Liszewski M. K. Strain L. Blouin J. Brown A. L. (2008). Mutations in complement C3 predispose to development of atypical hemolytic uremic syndrome. *Blood* 112 4948–4952. 18796626 10.1182/blood-2008-01-133702 PMC2597601

[B13] Gadsden M. H. Mcintosh E. M. Game J. C. Wilson P. J. Haynes R. H. (1993). dUTP pyrophosphatase is an essential enzyme in Saccharomyces cerevisiae. *EMBO J.* 12 4425–4431. 10.1002/j.1460-2075.1993.tb06127.x 8223452 PMC413740

[B14] Hamza A. Tammpere E. Kofoed M. Keong C. Chiang J. Giaever G. (2015). Complementation of yeast genes with human genes as an experimental platform for functional testing of human genetic variants. *Genetics* 201 1263–1274. 10.1534/genetics.115.181099 26354769 PMC4649650

[B15] Huh W. K. Falvo J. V. Gerke L. C. Carroll A. S. Howson R. W. Weissman J. S. (2003). Global analysis of protein localization in budding yeast. *Nature* 425 686–691. 14562095 10.1038/nature02026

[B16] Kachroo A. H. Laurent J. M. Yellman C. M. Meyer A. G. Wilke C. O. Marcotte E. M. (2015). Evolution. Systematic humanization of yeast genes reveals conserved functions and genetic modularity. *Science* 348 921–925. 10.1126/science.aaa0769 25999509 PMC4718922

[B17] Karaca E. Harel T. Pehlivan D. Jhangiani S. N. Gambin T. Coban Akdemir Z. (2015). Genes that affect brain structure and function identified by rare variant analyses of mendelian neurologic disease. *Neuron* 88 499–513. 10.1016/j.neuron.2015.09.048 26539891 PMC4824012

[B18] Li J. Gao B. Xiao X. Li S. Jia X. Sun W. (2016). Exome sequencing identified null mutations in LOXL3 associated with early-onset high myopia. *Mol. Vis.* 22 161–167. 26957899 PMC4764606

[B19] Maddirevula S. Alhebbi H. Alqahtani A. Algoufi T. Alsaif H. S. Ibrahim N. (2019a). Identification of novel loci for pediatric cholestatic liver disease defined by KIF12, PPM1F, USP53, LSR, and WDR83OS pathogenic variants. *Genet. Med.* 21 1164–1172. 10.1038/s41436-018-0288-x 30250217

[B20] Maddirevula S. Alzahrani F. Al-Owain M. Al Muhaizea M. A. Kayyali H. R. Alhashem A. (2019b). Autozygome and high throughput confirmation of disease genes candidacy. *Genet. Med.* 21 736–742. 10.1038/s41436-018-0138-x 30237576 PMC6752307

[B21] Maddirevula S. Kuwahara H. Ewida N. Shamseldin H. E. Patel N. Alzahrani F. (2020). Analysis of transcript-deleterious variants in Mendelian disorders: implications for RNA-based diagnostics. *Genome Biol.* 21:145. 10.1186/s13059-020-02053-9 PMC7298854 32552793

[B22] Monies D. Abouelhoda M. Assoum M. Moghrabi N. Rafiullah R. Almontashiri N. (2019). Lessons learned from large-scale, first-tier clinical exome sequencing in a highly consanguineous population. *Am. J. Hum. Genet.* 104 1182–1201. 31130284 10.1016/j.ajhg.2019.04.011 PMC6562004

[B23] Monies D. Maddirevula S. Kurdi W. Alanazy M. H. Alkhalidi H. Al-Owain M. (2017). Autozygosity reveals recessive mutations and novel mechanisms in dominant genes: implications in variant interpretation. *Genet. Med.* 19 1144–1150. 10.1038/gim.2017.22 28383543

[B24] Reuter M. S. Tawamie H. Buchert R. Hosny Gebril O. Froukh T. Thiel C. (2017). Diagnostic yield and novel candidate genes by exome sequencing in 152 consanguineous families with neurodevelopmental disorders. *JAMA Psychiatry* 74 293–299. 10.1001/jamapsychiatry.2016.3798 28097321

[B25] Richards S. Aziz N. Bale S. Bick D. Das S. Gastier-Foster J. (2015). Standards and guidelines for the interpretation of sequence variants: a joint consensus recommendation of the American College of Medical Genetics and genomics and the association for molecular pathology. *Genet. Med.* 17 405–424. 10.1038/gim.2015.30 25741868 PMC4544753

[B26] Rose M. D. Winston F. M. Hieter P. (1990). *Methods in Yeast Genetics: A Laboratory Course Manual.* Cold Spring Harbor, NY: Cold Spring Harbor Laboratory Press.

[B27] Salih M. A. Seidahmed M. Z. El Khashab H. Y. Hamad M. H. Bosley T. M. Burn S. (2015). Mutation in GM2A leads to a progressive chorea-dementia syndrome. *Tremor. Other. Hyperkinet. Mov.* 5:306 10.5334/tohm.246 PMC4502426 26203402

[B28] Schramm E. C. Roumenina L. T. Rybkine T. Chauvet S. Vieira-Martins P. Hue C. (2015). Mapping interactions between complement C3 and regulators using mutations in atypical hemolytic uremic syndrome. *Blood* 125 2359–2369. 10.1182/blood-2014-10-609073 25608561 PMC4392009

[B29] Shaheen R. Patel N. Shamseldin H. Alzahrani F. Al-Yamany R. ALMoisheer A. (2016). Accelerating matchmaking of novel dysmorphology syndromes through clinical and genomic characterization of a large cohort. *Genet. Med.* 18 686–695. 10.1038/gim.2015.147 26633546

[B30] Shamia A. Shaheen R. Sabbagh N. Almoisheer A. Halees A. Alkuraya F. S. (2015). Revisiting disease genes based on whole-exome sequencing in consanguineous populations. *Hum. Genet.* 134 1029–1034. 10.1007/s00439-015-1580-3 26141664

[B31] Sirr A. Lo R. S. Cromie G. A. Scott A. C. Ashmead J. Heyesus M. (2020). A yeast-based complementation assay elucidates the functional impact of 200 missense variants in human PSAT1. *J. Inherit. Metab. Dis.* 43 758–769. 10.1002/jimd.12227 32077105 PMC7444316

[B32] Stark R. Kibbey R. G. (2014). The mitochondrial isoform of phosphoenolpyruvate carboxykinase (PEPCK-M) and glucose homeostasis: has it been overlooked? *Biochim. Biophys. Acta Gen. Sub.* 1840 1313–1330. 10.1016/j.bbagen.2013.10.033 24177027 PMC3943549

[B33] Strande N. T. Riggs E. R. Buchanan A. H. Ceyhan-Birsoy O. Distefano M. Dwight S. S. (2017). Evaluating the clinical validity of gene-disease associations: an evidence-based framework developed by the clinical genome resource. *Am. J. Hum. Genet.* 100 895–906. 10.1016/j.ajhg.2017.04.015 28552198 PMC5473734

[B34] Sun S. Yang F. Tan G. Costanzo M. Oughtred R. Hirschman J. (2016). An extended set of yeast-based functional assays accurately identifies human disease mutations. *Genome Res.* 26 670–680. 10.1101/gr.192526.115 26975778 PMC4864455

[B35] Tchigvintsev A. Singer A. U. Flick R. Petit P. Brown G. Evdokimova E. (2011). Structure and activity of the Saccharomyces cerevisiae dUTP pyrophosphatase DUT1, an essential housekeeping enzyme. *Biochem. J.* 437 243–253. 10.1042/bj20110304 21548881 PMC4671291

[B36] Voth W. P. Richards J. D. Shaw J. M. Stillman D. J. (2001). Yeast vectors for integration at the HO locus. *Nucleic Acids Res.* 29:e59. 10.1093/nar/29.12.e59 PMC55758 11410682

[B37] Webb E. A. Almutair A. Kelberman D. Bacchelli C. Chanudet E. Lescai F. (2013). ARNT2 mutation causes hypopituitarism, post-natal microcephaly, visual and renal anomalies. *Brain* 136 3096–3105. 10.1093/brain/awt218 24022475 PMC3784281

[B38] Winston F. Dollard C. Ricupero-Hovasse S. L. (1995). Construction of a set of convenient Saccharomyces cerevisiae strains that are isogenic to S288C. *Yeast* 11 53–55. 10.1002/yea.320110107 7762301

[B39] Winzeler E. A. Shoemaker D. D. Astromoff A. Liang H. Anderson K. Andre B. (1999). Functional characterization of the S. cerevisiae genome by gene deletion and parallel analysis. *Science* 285 901–906. 10.1126/science.285.5429.901 10436161

[B40] Wiszniewski W. Gawlinski P. Gambin T. Bekiesinska-Figatowska M. Obersztyn E. Antczak-Marach D. (2018). Comprehensive genomic analysis of patients with disorders of cerebral cortical development. *Eur. J. Hum. Genet.* 26 1121–1131. 29706646 10.1038/s41431-018-0137-z PMC6057976

