# Supplementary material for: Exploiting the Autozygome to Support Previously Published Mendelian Gene-Disease Associations: An Update
Source: Front Genet. 2020 Dec 31;11:580484. doi: 10.3389/fgene.2020.580484 (PMC7806527; doi:10.3389/fgene.2020.580484)
Supplement: Supplementary Figure S1 — Pedigrees and segregation data of all the study families. [file Image_1.pdf]

Figure S1: The family pedigrees of 24 families. Homo: Homozygous; Het: Heterozygous; NT: Not Tested; WT: Wild type.

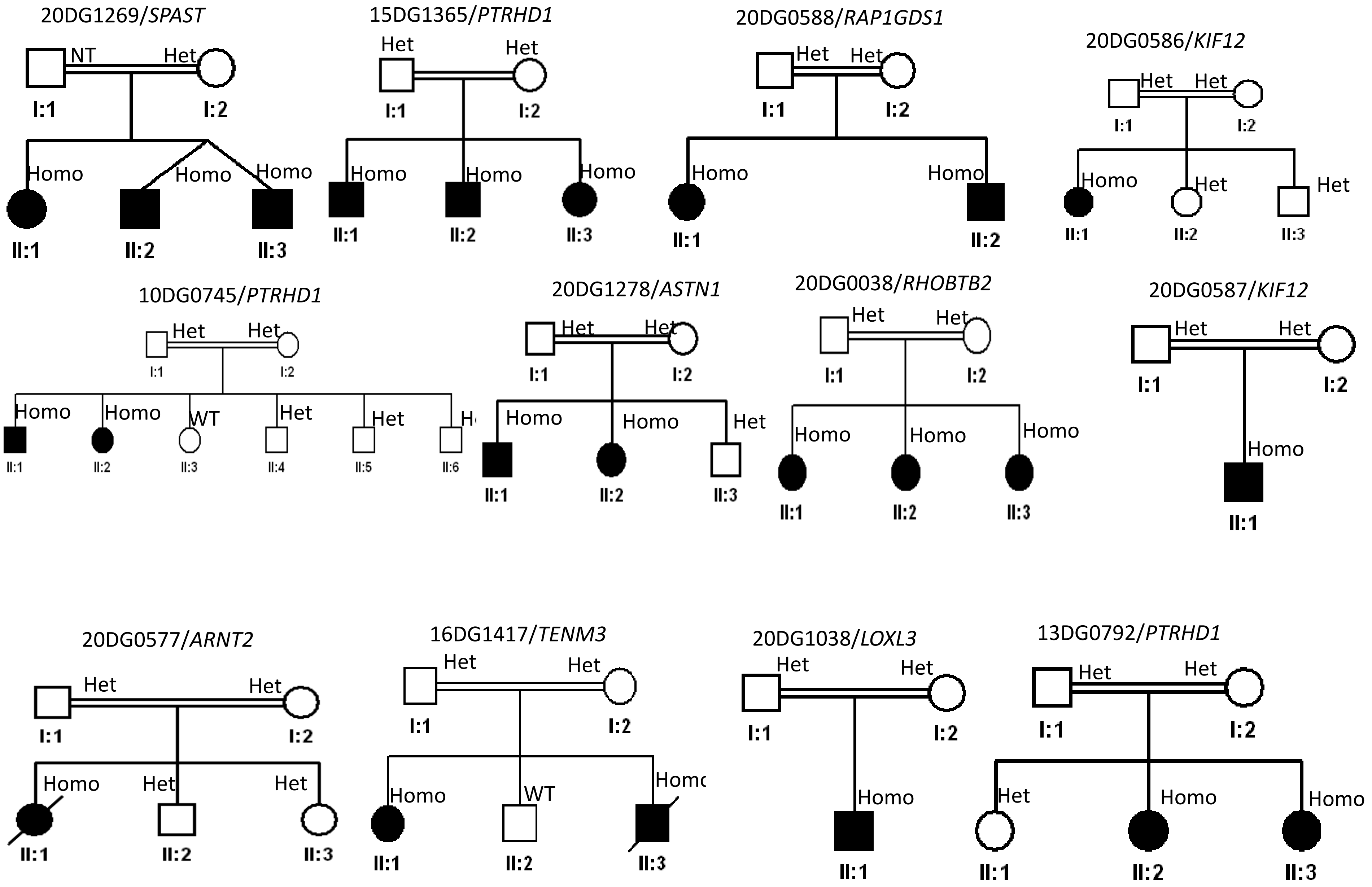

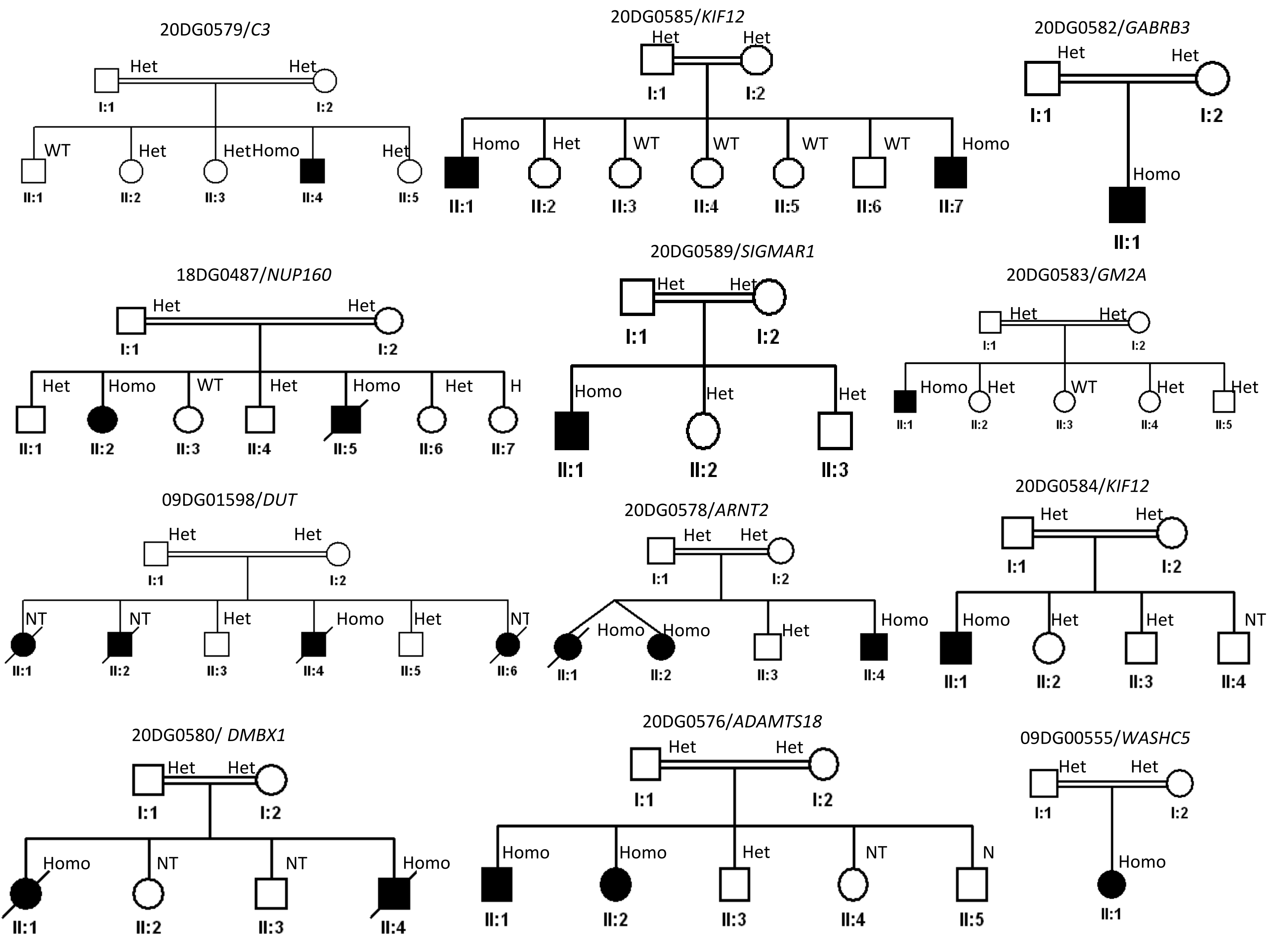
20DG0579/C3
Het
Het
I:1
I:2
WT
Het
HetHomo
Het
II:1
II:2
II:3
II:4
II:5
20DG0585/KIF12
Het
Het
I:1
I:2
Homo
Het
WT
WT
WT
WT
Homo
II:1
II:2
II:3
II:4
II:5
II:6
II:7
20DG0582/GABRB3
Het
Het
I:1
I:2
Homo
II:1
18DG0487/NUP160
Het
Het
I:1
I:2
Het
Homo
WT
Het
Homo
Het
H
II:1
II:2
II:3
II:4
II:5
II:6
II:7
20DG0589/SIGMAR1
Het
Het
I:1
I:2
Homo
Het
Het
II:1
II:2
II:3
20DG0583/GM2A
Het
Het
I:1
I:2
Homo
Het
WT
Het
Het
II:1
II:2
II:3
II:4
II:5
09DG01598/DUT
Het
Het
I:1
I:2
NT
NT
Het
Homo
Het
NT
II:1
II:2
II:3
II:4
II:5
II:6
20DG0578/ARNT2
Het
Het
I:1
I:2
Homo
Homo
Het
Homo
II:1
II:2
II:3
II:4
20DG0584/KIF12
Het
Het
I:1
I:2
Homo
Het
Het
NT
II:1
II:2
II:3
II:4
20DG0580/ DMBX1
Het
Het
I:1
I:2
Homo
NT
NT
Homo
II:1
II:2
II:3
II:4
20DG0576/ADAMTS18
Het
Het
I:1
I:2
Homo
Homo
Het
NT
N
II:1
II:2
II:3
II:4
II:5
09DG00555/WASHC5
Het
Het
I:1
I:2
Homo
II:1
