# Supplementary material for: Exploiting the Autozygome to Support Previously Published Mendelian Gene-Disease Associations: An Update
Source: Front Genet. 2020 Dec 31;11:580484. doi: 10.3389/fgene.2020.580484 (PMC7806527; doi:10.3389/fgene.2020.580484)
Supplement: Supplementary Table S4 — Clinical synopsis of the 24 patients described in this study. ACMG, American College of Medical Genetics; SHGP, Saudi Human Genome Program; HGMD, Human Gene Mutation Database. [file Table_4.docx]

| **Case ID** | **Genetic diagnosis and clinical description** | **Additional information (Variants)** |
| --- | --- | --- |
| 20DG0576 | *ADAMTS18 (*NM_199355.2:c.1298C>A;p.(Thr433Asn)/Homozygous)  The index is 18 year-old male, obese, known to have congenital renal abnormalities and hypertension with poor vision. Best corrected visual acuity is 20/40 in either eye. Ophthalmic evaluation showed microcornea, myopic chorioretinal atrophy, and telecanthus. His 11 year-old sister is obese with decreased vision since early childhood. Ophthalmic evaluation showed microcornea, myopic chorioretinal atrophy, and telecanthus. Best corrected visual acuity is 20/60 in either eye. Cycloplegic refraction is –18.00 -7.00X176 OD, -13.50 -7.00X 010 OS. Axial lengths are 30.83 mm OD and 28.74 mm OS; anterior chamber depths are 2.98 mm OD and 2.89 mm OS. Parents are first cousins with four healthy kids. | ACMG: Likely pathogenic (PM2;PP3;PP4;PP5;BP1)  SHGP (n=2379): Absent  HGMD: CM168291 |
| 20DG0577 | *ARNT2 (*NM_014862.3:c.147-1G>A/Homozygous)  A 4-year-old girl with feeding difficulty, developmental delay, microcephaly, generalized hypotonic and encephalopathic with squint, poor vision, obesity and history of seizures. The etiology of the seizure at this point is unclear and epilepsy in the recent past has been resolved. She was offered for the PEG tube insertion for the purpose of feeding. Apart from the above-mentioned issues, the CVS, respiratory, GIT, urogenital, musculoskeletal, and CNS were unremarkable. She is the product of consanguineous marriage with no family history and healthy siblings. | ACMG: Pathogenic (PVS1;PM2;PP3)  SHGP (n=2379): Absent  HGMD: CS1715456 |
| 20DG0578 | *ARNT2 (*NM_014862.3:c.147-1G>A/Homozygous)  Proband is a twin II with short stature, microcephaly. She is the product of 38 week, delivered by caesarean section due to previous sections, admitted no NICU because of hypoglycemia. Found to have congenital hear t disease (Small PDA, and VSD), she developed hypernatremic dehydration, at age of one month, after which she was diagnosed to have septo-optic dysplasia. She did not achieve normal development at any age in her life, tell 20 month of age, she was unable to control head, cannot rule over, cannot fix on objects, and had no speech. Her Brain MRI showed absence posterior part of pituitary and bilateral posteropatital subglial swelling. At age of 18 month she presented with acute attach of Meningoencephalitis, with decrease level of consciousness, and status epilepticus after febrile illness, managed in PICU. She died after cardiac arrest. Parents are firs cousin, Positive family history of GDD, IUGR, blindness, nystagmus, septo-optic dysplasia and hypopituitarism in two siblings and 2 cousins. | ACMG: Pathogenic (PVS1;PM2;PP3)  SHGP (n=2379): Absent  HGMD: CS1715456 |
| 20DG1278 | *ASTN1* (NM_001286164.2:c.3159_3160del;p.(Gln1053Hisfs*13)/Homozygous)  A 5 years old boy born to a healthy Saudi couple with autism, ADHD and seizures. EEG showed high voltage 6-7 hz/s, occasional focal sharp wave and he is on valproic acid. His brain MRI is unremarkable. Parents are firs cousins with family of autism and sub-normal mentality. Her sibling is also affected with autism, ADHD and ID with no seizures. | ACMG: Pathogenic (PVS1:PM1;PM2)  SHGP (n=2379): Absent  HGMD: Absent |
| 20DG0579 | *C3 (*NM_000064.2:c.3343G>A;p.(Asp1115Asn)/Homozygous)  A 3 year old Saudi male child diagnosed antenatally with a single left sided kidney who developed lethargy, vomiting, oliguria with renal failure, hypertension and seizure during early infancy necessitating hospitalization in a pediatric intensive care setting with mechanical ventilation for 1 month and peritoneal dialysis for about 3 months. He was diagnosed then with atypical hemolytic uremic syndrome with the following clinical findings of anemia, thrombocytopenia, hemolysis and uremia along with a positive family history for a first degree cousin (paternal side) with end stage renal disease s/p kidney transplantation graft loss secondary to atypical hemolytic uremic syndrome.  Both parents are first degree cousins on the paternal side. The patient received Eculizumab (anti C5 monoclonal antibody) since initial diagnosis till now with improvement in his renal function and getting off dialysis at 5 months of age. He remains on antihypertensive medications. Over the last 3 years, he had on 2-3 occasions reactivation of his hemolytic uremic syndrome with febrile illnesses while on Eculizumab, with the following laboratory studies abnormalities: hemoglobin down to 5 g/l, platelets down to 40,000, Creatinine up to 353 micromoles/l, LDH up to 1913 U/L, total bilirubin 55 micromoles/L and evidence of red cell fragments on peripheral blood morphology smear. The patient is on the 50^th^ percentile (weight-for-age) and on the 90^th^ percentile (stature-for-age) with a creatinine of 38 micromoles/L. | ACMG: Likely Pathogenic (PM1;PM2;PP3;PP5;BP1) Absent CM086785  SHGP (n=2379): Absent  HGMD: CM086785 |
| 20DG0580 | *DMBX1* (NM_147192.2:c.367C>T;p.(Arg123Trp)/Homozygous)  Index is 2 years old girl with hypotonia with global developmental delay born to healthy Saudi couple. Her Parent first cousin, they have 2 healthy, the mother had 2 fetal deaths of unknown cause. Her Seizure disorder controlled on carbamazepine. She is cared at home with moderate motor delay, developmental assessment at age of 1,6 years revealed she could be fixating and following. Socially, she was interacting with her parents able to sit. She had mild hypotonia with appendicular hypertonia with normal muscle bulk, no neurocutaneous stigmata or organomegaly. Her younger brother also similarly affected. Basic hematological, biochemical and metabolic work up were unremarkable (including plasm amino acid, lactate, ammonia, tandem MS, Urine Organic acid). Both are died with apnea and respiratory collapse. | ACMG: Likely pathogenic (PM1;PM2;PP3;PP5)  SHGP (n=2379): Absent  HGMD: CM150810 |
| 09DG01598 | *DUT (*NM_001025248.1:c.647G>A;p.(Arg216Gln)/Homozygous)  Index is 27month old boy with congenital dyserythropoietic anemia and pancytopenia. All his developmental mile stones are normal. Bone marrow analysis revealed acute erythropoiesis. No seizures and unremarkable metabolic profiling. He had significant family history of neonatal deaths. Parents are healthy and first cousins. | ACMG: Likely Pathogenic (PS3;PM2;PP3)  SHGP (n=2379): Absent  HGMD: Novel |
| 20DG0582 | *GABRB3* (NM_001191320.1:c.890C>G;p.(Ser297*)/Homozygous)  A 3 years old boy static encephalopathy and focal seizure left-sided with secondary generalization, neonatal onset with global developmental delay. He is a product of full term, normal spontaneous vertex delivery. Currently he can sit with minimal support, can hold fork. Brain MRI shown asymmetry of the temporal horn of the lateral ventricle in the form of the right temporal horn being larger than the left temporal horn, possibly indicating previous ictal focus. No evidence of mesial temporal sclerosis. Nonspecific subcortical high signal intensity and his EEG is unremarkable. No dysmorphic features, no neurocutaneous stigmata. He has extropia of the right eye, no ophthalmoplegia. Parents are first-degree cousins. He is the first child. No family history of epilepsy. | ACMG: Likely Pathogenic (PM1;PM2;PP2;PP3)  SHGP (n=2379): Absent  HGMD: Novel |
| 20DG0583 | *GM2A* (NM_000405.5:c.164C>T;p.(Pro55Leu)/Homozygous)  An 11-year-old boy with developmental regression, seizures and behavioral change since age of 6 years. Brain MRI revealed relative stability of mildly diffuse cerebral volume loss with no evidence of focal lesion or acute insult. He was doing fine in usual state of health until the age of 6½ years old when his mother noticed that he started to lose his motor skills, mild stone regression in form of unclear talking, not complete sentences then he lost fine motor, unable to eat or drink by himself with progressive loss of gross motor as well, unsteady gait and loss of hand skills with abnormal repetitive behavior in form of repeating word, inability to concentrate with poor eyes contact, screaming, talking to himself, visual hallucinations and excessive fear. He has cognitive decline with orofacial dyskinesia and loss of toilet training. There was no history of fever, trauma, or abnormal movement, no history of loss of consciousness, no respiratory or cardiac symptoms, no vomiting, change in bowel habit, or urinary symptoms, no skin rash or joint pain, no hearing problems, no swallowing problems. He also has a history of frequent episodes of behavioral arrest sometimes associated with facial twitching. Index is the only affected with four healthy siblings and he is the product of consanguineous parents. | ACMG: Likely Pathogenic (PM1;PM2;PP3;PP5;BP1)  SHGP (n=2379): Absent  HGMD: CM150802 |
| 20DG0584 | *KIF12* (NM_138424.1:c.610G>A;p.(Val204Met)/Homozygous)  A 14-year-old girl with congenital hepatic fibrosis, history of portal hypertension/esophageal varices and hypersplenism. She was born preterm with IUGR to consanguineous parents. Her ffamily history is significant for the death of elder sister due to hematemesis at the age of 14 years. Ultrasound liver transplant showed minimal diffuse increased transplanted liver parenchymal echogenicity with no focal lesions, intrahepatic biliary duct dilatation, prehepatic collection of fluid, normal patency of hepatic vessels, and moderate splenomegaly with no focal lesions. | ACMG: Likely pathogenic# (PM2, PP1, PP3, PP5)  SHGP (n=2379): Absent  HGMD: CM195983 |
| 20DG0585 | *KIF12* (NM_138424.1:c.463C>T;p.(Arg155*)/Homozygous)  A 10months old boy with failure to thrive and chronic liver disease (high gamma GT cholestasis) with very strong family history. Parents are first degree cousins. His ultrasound showed Mild worsening of the hepatomegaly with heterogeneous periportal liver echogenicity, likely representing an underlying liver parenchymal disease, probably of biliary etiology and mild splenomegaly. His biochemical profiles revealed elevated levels of Bilirubin, total (336.5 umol/L), ALT (50.6 U/L), AST (122.7 U/L), Alkaline Phosphatase (1,489.0 U/L) GGT (293 IU/L). | ACMG: Pathogenic (PVS1;PM2;PP3)  SHGP (n=2379): Absent  HGMD: CM196755 |
| 20DG0586 | *KIF12* (NM_138424.1:c.290A>G;p.(His97Arg) /Homozygous)  An 18-year-old female with liver cirrhosis of undetermined etiology. She was referred for consideration for liver transplantation because her sister also had liver disease and died because of advanced liver cirrhosis and related complications. She is currently compensated with no history of encephalopathy, ascites or GI bleeding. She is fully functional and mobile. She has no limitations in terms of communication ability or mobility. She is attending school and is doing reasonably well. She has no other comorbidities. Chest and cardiovascular examinations were unremarkable. Elevated levels of alkaline phosphatase and gamma GT are noticed. Abdominal examination revealed no ascites and she had no leg edema. She has no jaundice. Her echocardiogram was also unremarkable. CT scan showed shrunken cirrhotic liver with no focal liver lesions and her liver vessels are all patent. She has significant splenomegaly. Her autoimmune markers are also negative. | ACMG: VUS (PM2;PP3)  SHGP (n=2379): Absent  HGMD: Novel |
| 20DG0587 | *KIF12* (NM_138424.1:c.463C>T;p.(Arg155*)/Homozygous)  An 8 years old girl case of resolving high GGT cholestasis. She was admitted with us at the age of 2 months with jaundice started at 3 days of age. She was found to have high GGT cholestasis. History showed normal pregnancy with normal delivery and birth weight. She is a product of a consanguineous marriage with no family history of liver disease or neonatal deaths. Work up for biliary atresia was negative. Ophthalmologic and cardiac evaluation was normal. She continued follow up in OPD. She is thriving well (weight and height between 75th-90th centile) with no specific complaint or concern but she continued to have slightly high liver enzymes with normal synthetic and execratory functions. Latest laboratory work up was on October 2018 and showed: AST 105(68), GGT 90(69), Serum Bilie16 / 9.9, ALT 101(60), Albumin 39.1, VitD 52, ALP 646, PFIC III gene was negative. Parents are consanguineous.  Last abdominal ultra sound revealed static finding with diffuse coarsening of the liver parenchyma with nodular outline suggesting cirrhotic changes. Hepatic span measures 9.7 cm compared to previous measured 9.1 cm. No intra or extrahepatic bile duct dilatation. Gallbladder appears normal with echo-free lumen. The spleen is enlarged measures about 13.7 cm almost stable since previous exam, without focal lesions. Pancreas appears normal. No intra-abdominal free fluid. | ACMG: Pathogenic (PVS1;PM2;PP3)  SHGP (n=2379): Absent  HGMD: CM196755 |
| 20DG1038 | *LOXL3* (NM_032603.4:c.824dup;p.(Ala277Cysfs*57)/Homozygous)  Index is 13 years old boy with high myopia and history of retinal detachment.  He was born to a G1P0 25yr old mother following an uneventful pregnancy.  Delivery was NSVD at term.  His medical history is largely unremarkable otherwise except for struggling in 4th grade and he has mild ID with IQ 66. Chromosomal microarray was normal. Parents are first cousins. | ACMG: Pathogenic (PVS1;PM2;PP3)  SHGP (n=2379): Absent  HGMD: Novel |
| 18DG0487 | *NUP160* (NM_015231.1:c.1179+5G>A/Homozygous)  An 11 years old boy, known case of West syndrome, developmental delay and seizure on antiepileptics medications on regular follow up with Pediatric Neurology Clinic since November 2012. Controlled on valproic, vigabatrin and levocarnitine. He is spastic on wheelchair with intellectual disability with no dysmorphic features. His EEG reflects generalized cortical nonspecific dysfunction; and represents an encephalopathic stage, the presence of epilptiform discharges indicated partial seizure with or without generalization. In December 2016, he presented with 10 days history of periorbital edema and bilateral lower limb edema with no fever or change in LOC. Urine analysis showed marked proteinuria. He was diagnosed as nephrotic syndrome and started on prednisolone 60 mg daily for 6 weeks. He developed high blood pressure and was controlled on enalapril 4 mg o.d, amlodipine 5 mg o.d and Lasix 25 mg b.i.d. Due to his unresponsiveness to the steroids. He underwent renal biopsy, which showed features suggestive of focal segmental glomerulosclerosis FSGS collapsing variant. So, the dose of prednisone was tapered gradually until reaching 5 mg daily. He has a sister 19 years old with a similar condition Myoclonic seizure and focal segmental glomerulosclerosis. She developed end stage renal disease and underwent renal transplant. | ACMG: Pathogenic (PS3; PM2; PM4; PP1; PP3)  SHGP (n=2379): Absent  HGMD: Novel |
| 15DG1365 | *PTRHD1* (NM_001013663.1:c.365G>A;p.(Arg122Gln) /Homozygous)  Index is a 10 years old product of spontaneous vaginal delivery, full term with normal birth growth parameters, uneventful pregnancy but difficult labor so admitted in NICU fro couple of days. Parents are first cousin with two similar affected siblings. Since birth parents noticed that she was delayed in acquiring her millstones, hypotonia and hyperactivity. She sat at the age of 2 years and she walked at the age of 3 years but unsteady gait until known no speech. She is hyperactive, restless but no seizure. Family history positive for similar condition of two brothers in addition to one abortion. Clinical examination revealed normal growth parameters, facial dysmorphia inform of deep sited eyes hypertelorism, thick lips, prominent ears, hyperpigemnetd patch in right axilla, no organomegaly, power and tone were normal. No abnormal skeletal features, back examination was unremarkable. Hearing and visual assessment were unremarkable. Thyroid function test, vitamin D level, lipid profile, liver function test, were normal, neurometabolic screening test (amino acids, tandem MS, urine organic acids, ammonia, lactate) and basic genetic analysis, chromosomal analysis, FISH for anglmann syndrome, and chromosomal micro-array were all inconclusive. Brain MRI is unremarkable.  His elder brother is 11.10 years old product of spontaneous vaginal delivery, full term with normal birth growth parameters, uneventful pregnancy, during the first year of life parent noticed he is very active, restless, in addition to global developmental delay especially speech delay. He had first seizure of generalized tonic clonic with head tilting to left side, EEG was very abnormal with interictal epileptiform discharge. Clinical examination revealed height and weight within normal centile, subtle facial dysmorphia, normocephaly. His vision and hearing assessment were unremarkable, he is very hyperactive, restless, he follows commands after repeated asking to follow it, in addition to normal tone and power. Also no organomegaly or neurocutaneous stigmata. Thyroid function test, vitamin D level, liver function test, were normal, neurometabolic screening test (amino acids, tandem MS, urine organic acids, ammonia, lactate) and basic genetic analysis, chromosomal analysis, fragile X repeat and chromosomal micro-array were all inconclusive. Brain MRI is unremarkable. His younger sibling is 4.10 years old product of spontaneous vaginal delivery, full term with normal birth growth parameters, uneventful pregnancy. At age of one year he developed first attack of tonic clonic seizure. Parent noticed that he has global developmental delay special in speech. He has hyperactivity. His visual and hearing assessment were unremarkable. Developmental assessment at the 2 year of age showed he can have holed objects, he cannot follow commands, he can walk for few steps. Clinical examination showed normal growth parameters, no facial dysmorphia**,** and wood lamp examination revealed hypopigmentation macules in right side of neck, dorsum of left hand, right thigh. Systemic evaluation revealed normal tone and power, no organomegaly, skeletal examination was unremarkable. Thyroid function test, liver function test, were normal, neurometabolic screening test (amino acids, tandem MS, urine organic acids, ammonia, lactate) were unremarkable. Brain MRI/S showed bilateral frontoparietal prominent perivascular spaces, otherwise unremarkable MRI and MR spectroscopy of the brain. | ACMG: Likely Pathogenic (PM2;PP1;PP3;PP5;BP4)  SHGP (n=2379): 0.00546448 (8het)  HGMD: CM170768 |
| 10DG0745 | *PTRHD1* (NM_001013663.1:c.365G>A;p.(Arg122Gln) /Homozygous)  Index is a 19 years old male with non syndromic intellectual disability with the IQ 70 and aggressive behavior. No dysmorphic features with family history. His sister also similarly affected with non syndromic intellectual disability. Parents are first degree cousins with 6 healthy children. | ACMG: Likely Pathogenic (PM2;PP1;PP3;PP5;BP4)  SHGP (n=2379): 0.00546448 (8het)  HGMD: CM170768 |
| 13DG0792 | *PTRHD1* (NM_001013663.1:c.365G>A;p.(Arg122Gln) /Homozygous)  Index is a 4 years old girl with developmental delay and renal failure. Parents are consanguineous with the family history of similarly affected girl. She is dysmorphic with depressed nasal bridge and strabismus. Brain MRI revealed diffused cerebellar atrophy. Dual molecular diagnosis is observed in the patient 13DG0792, who is homozygous for the founder mutation PTRHD1 and a ciliopathy phenotype (Caroli disease) caused by a variant WDR35 (NM_001006657.1:c.206G>A; p.(Gly69Asp)). Parents are 3^rd^ degree cousins with a healthy child. | ACMG: Likely Pathogenic (PM2;PP1;PP3;PP5;BP4)  SHGP (n=2379): 0.00546448 (8het)  HGMD: CM170768 |
| 20DG0588 | *RAP1GDS1 (*NM_001100426.1:c.1444-1G>A /Homozygous)  Index is 4.8 years old girl with intellectual disability and microcephaly. She started walking at the age of three. At the age of 23 months her brain MRI shown abnormal intensity in the central segmental region suggested metabolic disorder. Here metabolic profiles are normal. Her younger also similarly affected. Both are dysmorphic. Parents are first cousins. | ACMG: Pathogenic (PVS1;PM2;PP3)  SHGP (n=2379): Absent  HGMD: Absent  PMID: 32431071 |
| 20DG0038 | *RHOBTB2 (*NM_001160036.2:c.460C>T:p.(Arg154*)/ Homozygous)  Index is a 4 year old girl with developmental delay since the age of 6 months, unsteady gait squint and she is a product of consanguineous parents (First cousins). She has another two younger sisters with similar clinical phenotype. Her paternal uncle and 2 paternal cousins have squint. Her brain MRI is unremarkable. At the age of 4months her eye examination revealed inward deviation of left eye. All growth parameters are normal. Her both sister with persistent intermittent ataxia (unsteadiness) when wake up in morning, developmental delay (speech delay), convergent squint. Three siblings are dysmorphic. | ACMG: Pathogenic (PVS1;PM2;PP3)  SHGP (n=2379): Absent  HGMD: Novel |
| 20DG0589 | *SIGMAR1* (NM_005866.2:c.73delG;p.(Val25Serfs*18) /Homozygous)  Index is an 18 year old male with spastic paraplegia since the age of 5. At the age of 10 years, he started to have recurrent falls and abnormal unsteady gait, mainly fall to the right side with increased fall frequency with running. The problem was mainly involving the lower limbs and no involvement of the upper limbs with no facial involvement. Patient feels his leg stiff with inward twisting of the right foot. Symptoms are continuous on a daily basis, with no specific aggravation or relieving factors. At the age of 16, he started to complain additional weakness of his hand, mainly the fingers, more pronounced on the right hand which makes it hard for him to lift heavy objects. No history of seizures, trauma. No dysphagia, dysarthria and diplopia. His brain MRI is unremarkable. Parents are first cousins with three health children. | ACMG: Pathogenic (PVS1;PM2;PP3)  SHGP (n=2379): Absent  HGMD: Novel |
| 20DG1269 | *SPAST* (NM_199436.2:c.1290A>T;p.(Lys430Asn)/Homozygous)  Index is a seven years old girl, born to a healthy Saudi couples. She presented with seizures, central hypotonia and regression of milestones. Currently she is unable to sit, can slightly roll over and depends on NGT feeding with no dysmorphic features. Her seizures are described as brief jerks lasting for 2- 3 seconds. Her EEG showed generalized spikes and wave and poly spikes. Her chromosomal array and brain MRI are unremarkable. Her two brothers are similarly affected. Parents are first cousins. | ACMG: Pathogenic  (PS1,PM1,PM2,PP2,PP3)  SHGP (n=2379):Absent  HGMD: Novel |
| 16DG1417 | *TENM3* (NM_001080477.1:c.6006_6009del;p.(Gln2003Phefs*10) /Homozygous)  Index is 6.5 years old Egyptian girl with microcornea and coloboma of optic disc. She is the product of full term pregnancy. Parents are healthy and first degree cousins. All her growth parameters are normal. Brain MRI and CT are unremarkable. A and B scan (ultrasound) of both eyes revealed distorted ocular contour, phakie globe with well delineated posterior capsule, acoustic evidence of choroidal coloboma, normal acoustic appearance of the optic nerve head shadow and acoustic evidence of optic nerve head cupping. She is the only affected with single healthy sibling. | ACMG: Pathogenic (PVS1;PM2;PP3)  SHGP (n=2379): Absent  HGMD: Novel |
| 09DG00555 | *WASHC5* (NM_014846.3:c.2849A>G;p.(Lys950Arg)Homozygous)  The index girl was evaluated at the age of 2.9 yrs with suspected dysmorphism.  She was born to a healthy G2P2+0 22yrs old Saudi mother. Parents are first cousins. Pregnancy was uncomplicated.  Growth parameters at delivery with weight of 2.1 kg and head circumference of 34 cm.  Neonatal history was unremarkable. Developmental history revealed that she walked at 9 months, she follows one step commands without gestures and has a vocabulary of 20 words.  She is dysmrophic with malar hypoplasia, micrognathia, mild turricephaly and broad hallux. CT skull confirmed the presence of lambdoid suture synostosis. Chromosomal microarray was normal. | ACMG: Likely Pathogenic (PM2;PP2;PP3 (Based on Varsome: Using strength Strong because this variant is predicted splicing (scSNV ADA Boost score = 0.999 is greater than 0.708) in gene WASHC5, for which loss-of-function is a known mechanism of disease (gene has 8 pathogenic LOF variants and LOF Z-Score = 3.29 is greater than 0.7), furthermore we have 8 pathogenic predictions from BayesDel_addAF, DANN, EIGEN, FATHMM-MKL, LIST-S2, M-CAP, MutationTaster and REVEL)  SHGP (n=2379): Absent  HGMD: Novel |
